# Diagnosis of central nervous system lymphoma via cerebrospinal fluid cytology: a case report

**DOI:** 10.1186/s12883-019-1317-3

**Published:** 2019-05-07

**Authors:** Hui Zhao, Miao Ma, Limin Zhang, Guanghui Zheng, Hong Lv, Jie Liu, Xiao Li, Bei Song, Guojun Zhang

**Affiliations:** 1 Laboratory Diagnosis Center, Beijing Tiantan Hospital, Capital Medical University, Beijing, 100050 China; 2 Beijing Engineering Research Center of Immunological Reagents Clinical Research, Beijing, 100050 China

**Keywords:** Central nervous system lymphoma, Cytology, Cerebrospinal fluid, CSF cytology

## Abstract

**Background:**

Primary central nervous system lymphoma (PCNSL) is the most prevalent brain, spinal cord, eyes, and leptomeningeal lymphoma. It is often misdiagnosed due to an unspecific presentation or unavailable biopsy and results in a poor prognosis. Although the craniocerebral imaging examination of PCNSL has some characteristics, it is limited, and atypical cases are especially difficult to identify with intracranial tumours and other diseases. The biopsy, as the gold standard for PCNSL diagnosis, is not eligible for all patients suspected of having PCNSL.

**Case presentation:**

This report documents a woman who presented with a three-month history of numbness and weakness in the right leg. She was treated with drugs at a local hospital for one month. She developed demyelination lesions and her symptoms were aggravated. The patient was admitted to the Department of Nerve Infection and Immunology at Tiantan Hospital. Head magnetic resonance imaging (MRI) enhanced scanning indicated significant inflammatory demyelinating disease, and lymphoma was not excluded. CSF revealed a high protein level and CSF cytology detected abnormal cells, PCNSL was eventually presumed according to positive CSF cytology and cytological detection of the cerebrospinal fluid flow.

**Conclusions:**

PCNSL is a highly invasive tumour. With the development of technologies such as cerebrospinal fluid cytology and flow cytology, CSF analysis has become one of the definite diagnosis methods, and the tumour cell finding in CSF is the only reliable basis for diagnosis. Flow cytometric analysis and gene rearrangement testing also provide objective evidence.

## Background

Lymphoma is a malignant tumour of the lymphohaematopoietic system that can affect all of the tissues and organs throughout the entire body. It is divided into two categories: non-Hodgkin lymphoma (NHL) and Hodgkin lymphoma (HL). Primary central nervous system lymphoma (PCNSL) is the most prevalent brain, spinal cord, eyes, and leptomeningeal lymphoma. Most PCNSL is non-Hodgkin lymphoma, which originates from B lymphocyte monoclonal proliferation [1]. Misdiagnosis is often due to the inspecificity of clinical presentation or inability to perform biopsy. This article introduces a case of central nervous system lymphoma diagnosed via morphological detection of the cerebrospinal fluid and confirms the importance of laboratory examination of cerebrospinal fluid for the diagnosis and differential diagnosis of this disease.

## Case presentation

A 54-year-old woman with symptoms of numbness and weakness in the right leg with no obvious cause presented to the field hospital. The diagnosis was the possibility of metastatic tumour after head CT and MRI examination. Pathological findings at another hospital suggested demyelinating disease. The patient was treated with hormone drugs in the field hospital and her symptoms were relieved. But the numbness in her right leg was aggravated, and symptoms of dizziness and nausea appeared two months later. The patient was eventually admitted to the Department of Nerve Infection and Immunology at Tiantan Hospital for concentric sclerosis. The patient had a history of hypertension, hyperthyroidism, and mammary gland hyperplasia but no history of diabetes, heart disease, hepatitis, tuberculosis, or drug allergy.

Physical examination after admission found a decrease in her calculation capacity. She had instability of pointing at her nose with her right finger and the tibial experiment with her knee in the nervous system examination. She had no other obvious abnormalities.

Laboratory examination after admission found that her leukocyte level in a routine blood examination was 13.52 × 10^9^/L. Her glucose (2.93 mmol/L), sodium (124 mmol/L), and chlorine (87 mmol/L) levels were decreased. Her fibroproteinogen (1.62 g/L) level was decreased. A routine CSF examination revealed her CSF had a yellow and clear appearance, and the total number of CSF cells was 514/μl. The number of leukocytes was 14/μl. Her CSF protein was high (82.54 mg/dl), and her 24-h IgG intrathecal synthesis rate was increased (13.54). The IgG oligoclonal band of her CSF was negative. Her cytokine interleukinin-10 level was 641.00 pg/ml .

The patient’s cytomegalovirus IgG of the CSF was positive. Neuronal antigen spectrum antibody IgG (CSF and blood) were negative. Tumour markers (female) were negative. Autoimmune antibody tests and protein electrophoresis were normal. The result of a CSF culture indicated Bacillus (suspected contamination). CSF gram stain, acid fast stain, and ink stain were negative (Tables 1, 2, 3, 4, 5).

**Table 1:** Blood routine

Test	Results	Unit	Reference range
leukocyte	14.33	10^9^/L	4.00–10.00
lymphocyte	3.51	10^9^/L	0.90–5.20
monocytes	0.47	10^9^/L	0.16–1.00
neutrophils	10.31	10^9^/L	2.00–7.50
eosinophils	0.03	10^9^/L	0–0.80
basophils	0.01	10^9^/L	0–0.20
erythrocyte	4.38	10^12^/L	3.50–5.50
hemoglobin	142	g/L	110–160
hematocrit	37.9	%	37.0–50.0
mean corpuscular volume	86.5	fl	80.0–100.0
mean Corpuscular hemoglobin	32.4	pg	27.0–32.0
mean corpuscular hemoglobin concentration	375	g/L	320–360
erythrocyte distribution width SD	41.1	fl	
erythrocyte distribution width CV	13	%	10.1–16.0
platelet	227	10^9^/L	100–300
platelet distribution width	10.1	fl	15.0–17.0
mean platelet volume	9.5	fl	7.0–11.0
large platelet ratio	20.3	%	
Platelet hematocrit	0.22	%

**Table 2:** CSF routine

Item	Test results	Unit	Reference range
CSF appearance	yellow and clear		colorless and clear
PandyT	positive		negative
Total CSF cells	514	/ul	0
Leukocyte	14	/ul	0

**Table 3:** CSF biochemistry

Item	Test results	Unit	Reference range
CSF sugar	4.49	mmol/L	2.50–4.50
CSF protein	82.54	mmol/L	15.00–45.00
CSF chloride	117	mmol/L	118–132

**Table 4:** IgG synthesis rate in the sheath of CSF for 24 h

Item	Test results	Unit	Reference range
IgG synthesis rate in the sheath of CSF	13.54		
CSF albumin	0.66	mg/ml	0–0.15
serum albumin	39.3	mg/ml	0–41.89
CSF IgG	0.093	mg/ml	0–0.019
serum IgG	9.03	mg/ml	0–11.486

**Table 5:** Immunofixation electrophoresis

Item	Test results	Reference range
IgG oligoclonal band of CSF	weakly positive	negative
IgG oligoclonal band of Blood	weakly positive	negative
specific oligoclonal band of CSF	negative	negative

Colour Doppler ultrasound of the patient’s abdomen, urinary system, thyroid, lower extremity veins, and superficial lymph nodes were performed, with no abnormal findings. PET-CT indicated no metastatic tumour lesions.

**Fig. 1:**
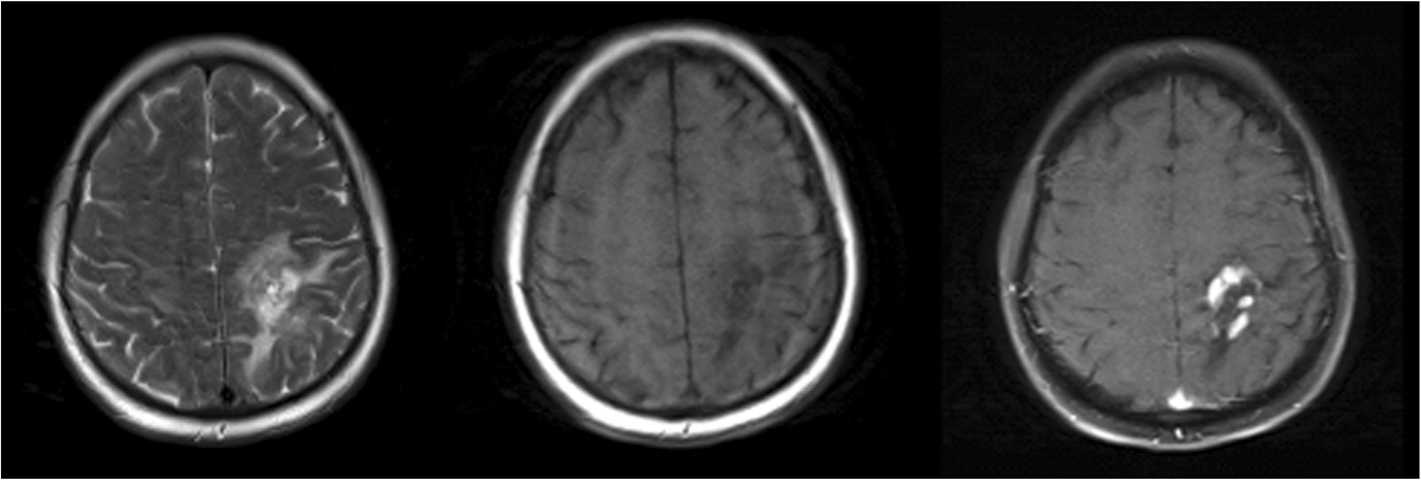
Head MRI enhanced scanning

Head MRI enhanced scanning demonstrated multiple abnormal signals in the double frontal cortex, subcortical, basal ganglia, temporal lobe, and callosum. Inflammatory demyelinating disease was more likely, and lymphoma was not excluded. Abnormal signals of the left parietal lobe were demonstrated after biopsy, and there was an ischaemic infarct in the patient’s right cerebellar hemisphere (Fig. 1).

**Fig. 2:**
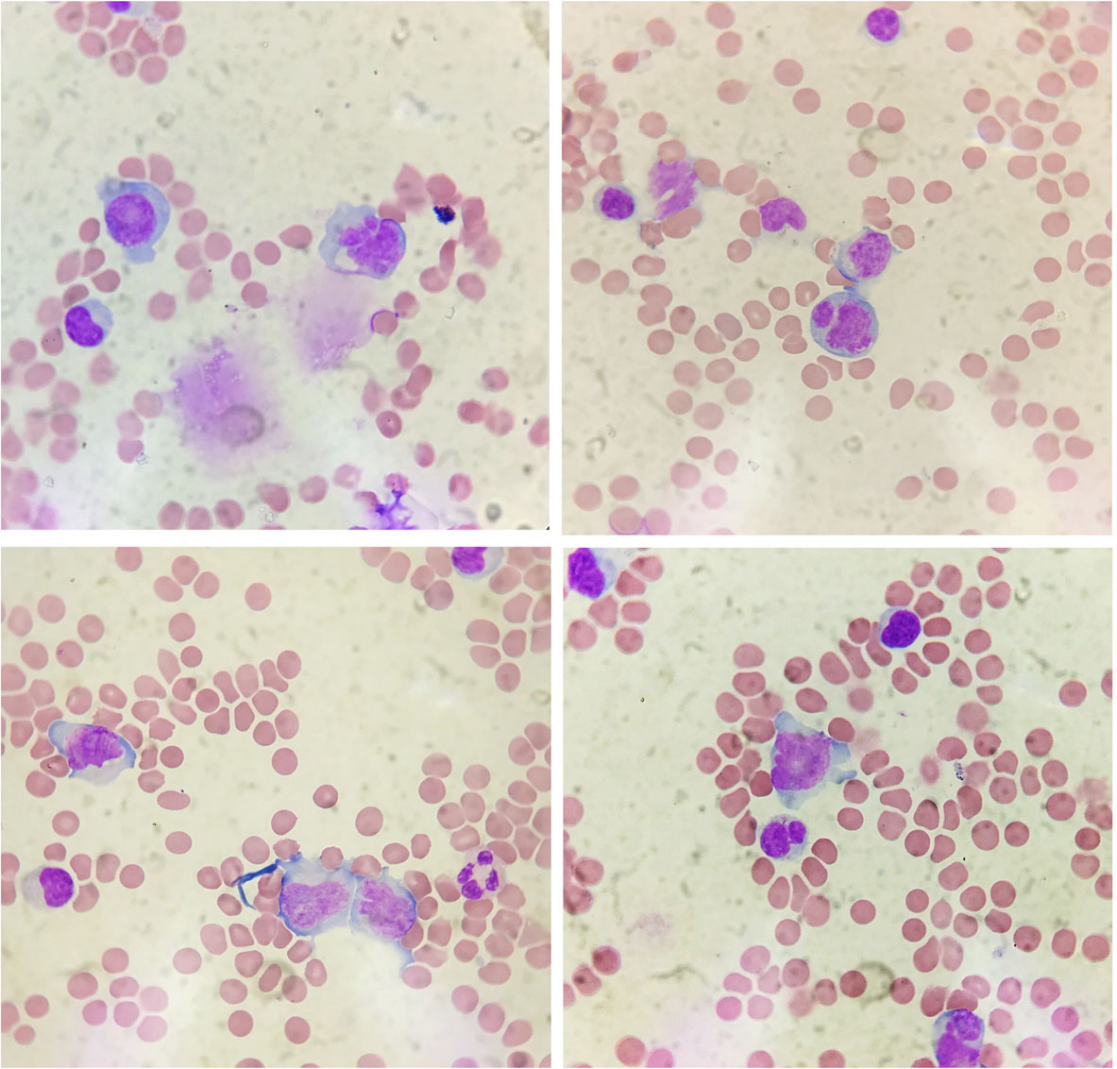
Cytology of CSF

CSF cytology conducted in the laboratory at Tiantan Hospital showed that a group of cells were suspected lymphoma cells. They had larger bodies and irregular nuclei, visible nucleolus, abundant cytoplasm, and deep staining, and this type of cell accounted for 11% (Fig. 2). We promptly communicated with the clinic, and the doctors were very excited about the valuable diagnostic basis of this difficult case.

**Fig. 3:**
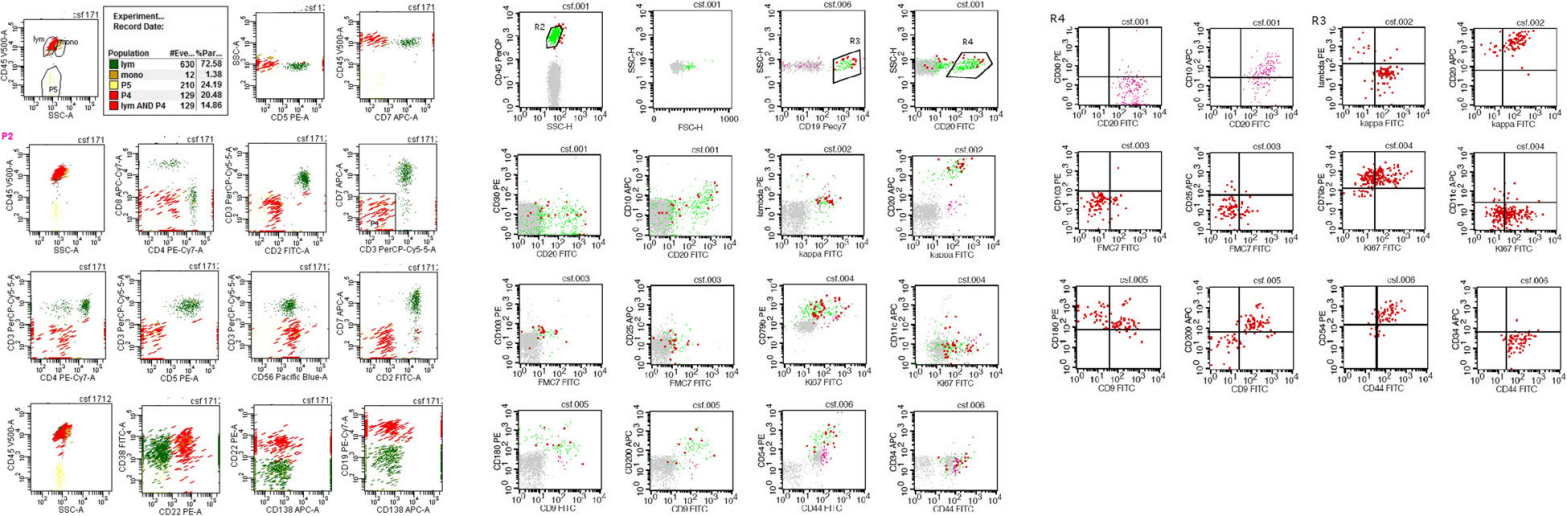
Flow cytometry of CSF

**Fig. 4:**
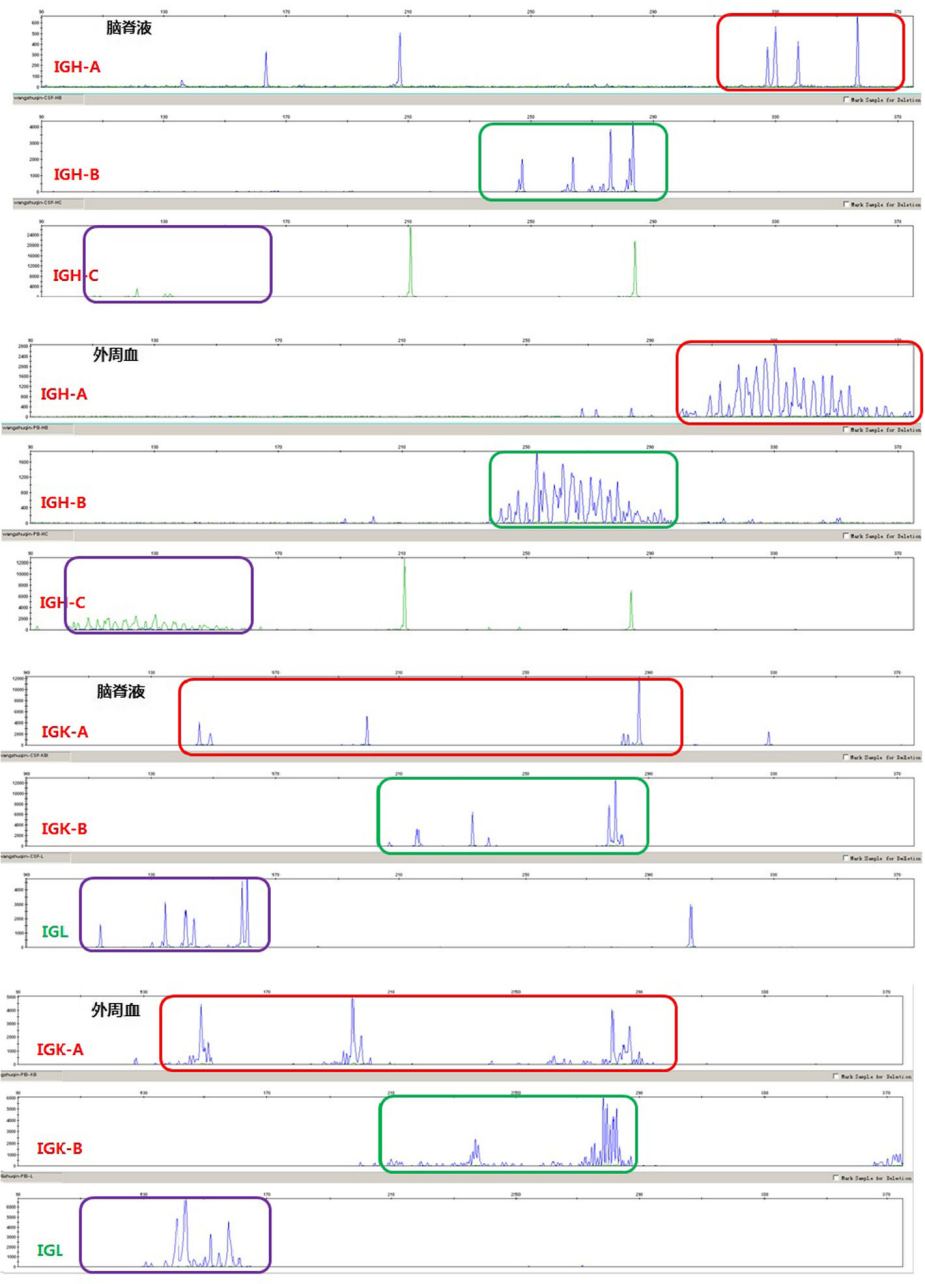
DNA sequencing of CSF

According to the CSF cytology results, the patient’s clinicians immediately prescribed flow cytometry and CSF DNA sequencing. CSF flow cytometry showed that 14.86% of her cells (129 karyocytes) expressed CD38, CD22, CD19, CD20, kappa, CD79b, CD180, CD54, and CD44. Some cells were considered malignant monoclonal mature B cells. They expressed K167 (40%), CD9, and CD200 and did not express CD56, CD2, CD7, CD3, CD4, CD8, CD138, CD30, lambda, FMC7, CD103, CD25, CD11C, and CD34 (Fig. 3). CSF DNA sequencing demonstrated that the IGH and IGL clonal rearrangement was negative and the IGK clonal rearrangement was positive (Fig. 4).

Combined with CSF cytology, flow cytometry, and DNA sequencing, the patient was diagnosed with primary central nervous system lymphoma (diffuse large B cell lymphoma) and transferred from the Department of Nerve Infection and Immunology to the Department of Haematology for further treatment.

In the Department of Haematology, the initial treatment was rituximab combined with a chemotherapy regimen (R-MAD). On the second day, the patient was administered a lumbar puncture and intrathecal injection of 50 mg cytarabine and 5 mg of dexamethasone, and then the routine, biochemical, and cytological examinations were repeated. CSF cytology showed that the lymphoma cells had decreased to 1%. On the third day, the patient had a clear state of mind but her spirit was weak, and there was no nausea and vomiting and no obvious adverse reactions to chemotherapy.

## Discussion and conclusions

Primary central nervous system lymphoma (PCNSL) is the most prevalent brain, spinal cord, eyes, and leptomeningeal lymphoma. Most PCNSL is non-Hodgkin lymphoma, which originates from B lymphocyte monoclonal proliferation. Its morphological and pathological features are similar to those of diffuse large B cell lymphoma (DLBCL). The WHO’s (2008) classification of haemopoietic and lymphatic tissue tumours classified DLBCL as an independent category originating in the central nervous system (CNS) [1].

PCNSL accounts for 3% of brain tumours, and more than 95% of cases are DLBCL, occurring in 50~70-year-old patients. The time from onset to presentation is within 2~3 months. The main symptoms of PCNSL are changes in mental state and intracranial hypertension such as headache, nausea and vomiting, papilledema, and local compression symptoms, including epilepsy, memory loss, unstable gait, visual impairment, blurred speech, and mild haemiplegia. In addition to brain damage, 10%~ 20% of patients have eye damage characterised by blurred vision or floating objects [2]. Because of the multifocal characteristics, the clinical manifestations of lymphoma patients may vary, making the disease difficult to diagnose [3]. Although the craniocerebral imaging examination of PCNSL has certain unique characteristics, it is limited, and atypical cases are especially difficult to distinguish from intracranial tumours and other diseases [4]. Although stereotactic biopsy can be clearly diagnosed, biopsy may lead to bleeding or more serious complications, especially in lesions around the brain stem. In addition, with the use of glucocorticoid before examination, the detection rate is significantly reduced. Therefore, the diagnosis of PCNSL is challenging, and most cases are misdiagnosed [5, 6].

PCNSL is a highly invasive tumour. It tends to spread along the CSF with the changing of the CSF composition or the shedding of tumour-causing cells into the CSF. Therefore, the CSF analysis has become one of the most definitive diagnostic methods. Tumour cell findings in CSF are the only reliable basis for the diagnosis, and 80% of patients with PCNSL have leptomeningeal involvement. PCNSL is described in *Neurological Clinical Cerebrospinal Fluid Cytology* by Su Xiuchu and Kong Fanyuan. This report documented a large number of atypical lymphocytes in the CSF of patients with central nervous system lymphoma. Atypical cells have small strong basophilic and blue cytoplasm around the nucleus and no particles. The nuclei are round or oval and often twisted, with chromatin clusters, visible nucleolus, and often mitotic phases. The tumour cells are sometimes large and irregular in shape. The amount of cytoplasm is more or medium with vacuoles, and the nucleus has a pea-like shape. Lymphoma cells in the CSF of PCNSL patients should be distinguished from activated lymphocytes. In general, lymphoma cells have irregular nuclei that are large and obvious, and vacuoles are common in the cytoplasm, but the activated lymphocytes demonstrate no signs of malignant cells.

Immunological methods contribute to the identification of lymphoma cells and activated lymphocytes. B cell lymphoma is the main type of lymphoma, and activated lymphocytes caused by infection account for a considerable proportion of T cells [7]. Therefore, the detection rate of malignant lymphoma can be improved using flow cytometry. Flow cytometry is an important diagnostic method for many haematologic malignancies. The advantage of this method is that the number of sample cells needed is small and the lymphoma cells and reactive lymphocytes can be distinguished according to the analysis of the cell size, complexity of the intracellular particles, and the presence of surface antigens. A retrospective study of 35 cases of lymphoproliferative disease involving the CNS showed that combined with flow cytometric immunophenotype and cytopathology, the detection rate of CSF increased by 50% compared with cytopathology alone [1]. Therefore, clinicians should exercise caution when relying only on cytology to diagnose PCNSL. If flow cytometry is combined with cytology and consistent results are obtained, the conclusions can be supported by reliable laboratory evidence. The use of monoclonal molecular markers, such as immunoglobulin gene rearrangement, can improve the positive diagnostic rate. In addition, the detection of antithrombin, soluble CD27, immunoglobulin light chains, and cytokine IL-10 in the CSF can assist with the diagnosis of PCNSL. The high expression of MYC, Bcl-2, and Bcl-6 genes in tumour tissues may indicate a poor prognosis [8, 9].

Cytological detection of the cerebrospinal fluid may have high specificity in the diagnosis of PCNSL. However, due to the spread range of PCNSL, CSF volume, and other factors, lymphoma cells might be undetectable in some of PCNSL cases. Thus, this method has certain limitations. In routine practise, our laboratory staff find it easier to obtain first-hand information than clinicians. Clinicians should actively communicate when diagnostic indications are present to promote further examinations for an accurate diagnosis and prompt treatment.

## Abbreviations

CD: Cluster of differentiation
CNS: Central nervous system
CSF: Cerebrospinal fluid
HL: Hodgkin lymphoma
IgG: Immunoglobulin G
MRI: Magnetic Resonance Imaging
NHL: Non Hondgkin lymphoma
PCNSL: Primary central nervous system lymphoma
PET-CT: Positron emission tomography computed tomography
R-MAD: Rituximab combined with chemotherapy regimen
